# Evaluating Tissue-Specific Recombination in a *Pdgfrα-CreER^T2^* Transgenic Mouse Line

**DOI:** 10.1371/journal.pone.0162858

**Published:** 2016-09-14

**Authors:** Megan O’Rourke, Carlie L. Cullen, Loic Auderset, Kimberley A. Pitman, Daniela Achatz, Robert Gasperini, Kaylene M. Young

**Affiliations:** 1 Menzies Institute for Medical Research, University of Tasmania, Liverpool Street, Hobart, Tasmania 7000, Australia; 2 School of Medicine, University of Tasmania, Liverpool Street, Hobart, Tasmania 7000, Australia; Massachusetts General Hospital/Harvard Medical School, UNITED STATES

## Abstract

In the central nervous system (CNS) platelet derived growth factor receptor alpha (PDGFRα) is expressed exclusively by oligodendrocyte progenitor cells (OPCs), making the *Pdgfrα* promoter an ideal tool for directing transgene expression in this cell type. Two *Pdgfrα-CreER*^*T2*^ mouse lines have been generated for this purpose which, when crossed with cre-sensitive reporter mice, allow the temporally restricted labelling of OPCs for lineage-tracing studies. These mice have also been used to achieve the deletion of CNS-specific genes from OPCs. However the ability of *Pdgfrα-CreER*^*T2*^ mice to induce cre-mediated recombination in PDGFR*α*^+^ cell populations located outside of the CNS has not been examined. Herein we quantify the proportion of PDGFRα^+^ cells that become YFP-labelled following Tamoxifen administration to adult *Pdgfrα-CreER*^*T2*^::*Rosa26-YFP* transgenic mice. We report that the vast majority (>90%) of PDGFRα^+^ OPCs in the CNS, and a significant proportion of PDGFRα^+^ stromal cells within the bone marrow (~38%) undergo recombination and become YFP-labelled. However, only a small proportion of the PDGFRα^+^ cell populations found in the sciatic nerve, adrenal gland, pituitary gland, heart, gastrocnemius muscle, kidney, lung, liver or intestine become YFP-labelled. These data suggest that *Pdgfrα-CreER*^*T2*^ transgenic mice can be used to achieve robust recombination in OPCs, while having a minimal effect on most PDGFRα^+^ cell populations outside of the CNS.

## Introduction

The platelet-derived growth factor receptor (PDGFR) was first identified in 1982, as a protein expressed by fibroblasts and arterial smooth muscle cells [1]. It was shown to facilitate normal growth and development by regulating critical cell processes including proliferation and differentiation [2–7], and mutations in this receptor were strongly associated with tumour growth [8–10]. In 1988 it was discovered that PDGFR was actually two receptors, named PDGFRα and PDGFRβ, that bind dimers of the PDGFs with different affinities [11]. PDGFRα is capable of binding all PDGFs except PDGF-DD [11,12], but has a strong affinity for the PDGF-A homodimer [13]. In the central nervous system (CNS), PDGFRα is selectively expressed by oligodendrocyte progenitor cells (OPCs) [14], and its activation by PDGF-AA has been shown to regulate the proliferation, migration and differentiation of this cell type in normal development as well as in response to demyelination [15].

The high specificity of PDGFRα expression by OPCs in the CNS, had made the *Pdgfrα* gene promoter an ideal tool to use in order to manipulate gene expression exclusively in OPCs without affecting other CNS cell types. For example, Rivers *et al*. [16] generated a bacterial artificial chromosome (BAC) transgenic mouse line, the *Pdgfrα-CreER*^*T2*^ transgenic mouse, which expresses Cre recombinase fused to the oestrogen-receptor type II, under the control of the *Pdgfrα* promoter. Tamoxifen administration to adult *Pdgfrα-CreER*^*T2*^::*Rosa26-YFP* transgenic mice resulted in ~50% of the OPCs in the brain [17], ~40% of the OPCs in the spinal cord and ~20% of OPCs in the optic nerve being labelled with yellow fluorescent protein (YFP) [18]. A second BAC transgenic *Pdgfrα-CreER*^*T2*^ mouse line was subsequently developed by Kang *et al*. [19], which achieves cre-mediated gene recombination in ≥ 90% of OPCs in the brain and spinal cord [19]. Both *Pdgfrα-CreER*^*T2*^ mouse lines have been widely used to label OPCs and trace their progeny *in vivo*. These studies largely report that OPCs continually generate new myelinating oligodendrocytes in the mature healthy CNS, and a small number indicate that OPCs differentiate into astrocytes (or astrocyte-like cells) and even Schwann cells in the CNS under certain pathological conditions [20].

More recently the *Pdgfrα-CreER*^*T2*^ transgenic mouse line produced by Rivers *et al*. [16] was used to conditionally ablate *myelin regulatory factor* from OPCs [21]. *Myelin regulatory factor* is not widely expressed outside of the CNS, which reduced the likelihood that this strategy would inadvertently affect the function of PDGFRα^+^ cell populations outside of the CNS. However, when using the *Pdgfrα-CreER*^*T2*^ transgenic mouse line to conditionally delete genes with a less discrete expression pattern, this would be an important consideration. To assess the ability of *Pdgfrα-CreER*^*T2*^ transgenic mice to induce recombination in PDGFRα^+^ cells within and outside of the CNS, we crossed *Pdgfrα-CreER*^*T2*^ *transgenic mice* [19] with *Rosa26-YFP* transgenic mice [22] and administered Tamoxifen to adult *Pdgfrα-CreER*^*T2*^::*Rosa26-YFP* offspring. The pattern of YFP labelling was then examined in a variety of tissues. We report that *Pdgfrα-CreER*^*T2*^ transgenic mice are highly suitable for OPC-directed gene recombination in the CNS, can be used to achieve robust recombination in OPCs, induce moderate recombination in PDGFRα^+^ bone marrow stromal cells, and have a minimal effect on other PDGFRα^+^ cell populations.

## Materials and Methods

### Transgenic Mice

*Pdgfrα-CreER*^*T2*^ transgenic mice [19] and *Rosa26-YFP* mice [22] were obtained from Jackson Laboratories. Male (n = 3) and female (n = 3) mice were used for this study. Mice were weaned at P20 and housed with gender matched littermates in individually ventilated cages. Food and water were available *ad libitum*. All experiments were approved by the University of Tasmania Animal Ethics Committee (13741).

### Genotyping

Genotyping was performed as previously described [23]. Ear biopsies were digested in 100mM Tris-HCl / 5mM EDTA / 200mM NaCl / 0.2% SDS / 0.48mg/ml proteinase K (ThermoFisher Scientific catalog number; AM2542). The cellular and histone proteins were precipitated by exposure to 6M Ammonium Acetate (Sigma; A1542) and incubation on ice. After centrifugation, the DNA was precipitated from the supernatant by exposure to isopropyl alcohol (Sigma; I9516), washed in 70% Ethanol (Sigma; E7023), resuspended in sterile MilliQ water and used as template DNA to genotype the mice by polymerase chain reaction (PCR). The PCR was performed as a 25μL reaction containing 50-100ng DNA, 0.5μL of each primer (100nmol/mL; GeneWorks) and 12.5 μL GoTaq^®^ green master mix (Promega) in MilliQ water. To genotype mice expressing the *Rosa26-YFP* transgene we used three primers: Rosa26 wildtype 5’ AAAGTC GCTCT GAGTT GTTAT, Rosa26 wildtype 3’ GGAGC GGGAG AAATGG ATATG and Rosa26 YFP 5’ GCGAA GAGTT TGTCC TCAACC in a program of: 94°C 4’, and 37 cycles of 94°C for 30”, 60°C for 45”, and 72°C for 60”, followed by 72°C for 10 minutes. The *Rosa26-YFP* PCR amplified a 550bp product corresponding to expression of the wildtype *Rosa26* gene and a 250bp product corresponding to the insertion of YFP into the *Rosa26* gene locus. The PCR designed to detect expression of the gene coding for Cre recombinase produced a 500bp product in the presence of Cre and no product when Cre was absent. The Cre PCR was carried out using the following primers: Cre 5’ CAGGTC TCAGG AGCTA TGTCC AATTT ACTGA CCGTA; Cre 3’ GGTGTT ATAAG CAATC CCCAGAA under the following conditions: 94°C for 4’, followed by 34 cycles of 94°C for 30”, 62°C for 45”, and 72°C for 60”, and a final 10 minutes at 72°C.

### Tamoxifen administration

Tamoxifen (Tx; Sigma) was reconstituted to 40 mg/ml in corn oil and sonicated for ≥ 1 hour until dissolved. Mice received a dose of 300mg/kg Tamoxifen by oral gavage, administered daily for 4 consecutive days from postnatal day 57 (P57). Mice were perfusion fixed with 4% paraformaldehyde (w/v) in PBS, 7 days after the initial dose (P63). Mice were weighed and monitored daily. No side-effects of Tamoxifen administration were observed. Our dosing regime provides the maximal amount of Tamoxifen that can be tolerated by young adult mice without observing side-effects such as weight loss [16]. By examining the tissue 3 days after the final Tamoxifen dose (7 days after the first dose) we allow recombination to occur and the fluorescent reporter to be expressed. This timeframe is sufficient to achieve stable labelling of a target cell population while minimizing the opportunity for labelled cells to proliferate or differentiate [16].

### Tissue preparation for histology

After perfusion fixation the brains were removed, sliced into 2mm thick coronal slices using a brain matrix, and immersion fixed for 90 min at room temperature. All other tissue was removed and immersion fixed for 90 min at room temperature. Tissue was cryopreserved in 20% sucrose (w/v) in PBS overnight at 4°C prior to embedding in optimal cutting temperature cryomatrix (Thermo Scientific) and storage at -80°C.

### Immunohistochemistry

Cryosections (30μm) were collected as floating sections from brain, spinal cord, eye, spleen, liver, kidney, heart, adrenal gland and pituitary gland. 30μm sections from sciatic nerve, gastrocnemius, intestine, and bone marrow (tail) were collected directly on to glass slides. Sections were processed for immunohistochemistry as previously described [24], using the following primary antibodies: goat anti-PDGFRα (1:200; GeneTex, California, USA), rat anti-GFP (1:2000; Nacalai Tesque, Kyoto, Japan; 04404–84), rabbit anti-S100β (1:500; Dako Australia Pty. Ltd., Campbellfield, Australia) and mouse anti-S100β (1:500; Sigma). Protein expression was visualised by secondary antibodies conjugated to Alexa Fluor-488, -568, -594 or -637 (Invitrogen): donkey anti-rat (1:500), donkey anti-goat (1:500), donkey anti-rabbit (1:500), donkey anti-mouse (1:500). Tissue was also exposed to Hoechst 33342 (Invitrogen; 1:10,000 dilution) to visualise the nuclei. Floating sections were mounted onto glass slides and the fluorescence preserved by the application of fluorescent mounting medium (Dako Australia Pty. Ltd., Campbellfield, Australia).

### Microscopy

Confocal images were collected using an UltraView confocal microscope with Volocity Software (Perkin Elmer, Massachusetts, USA) with standard excitation and emission filters for DAPI (Hoechst 33342), FITC (Alexa Fluor-488), TRITC (Alexa Fluor-568) and far red (Alexa Fluor-647). Hoechst was used to consistently define the region of interest within the tissue section, before a single z plane image was collected using the 20x objective, and stitched together using Volocity software. Cell counts were performed manually from exported images, viewing the images in ImageJ software version 1.46r (NIH, Washington DC, USA) and Adobe Photoshop CS6. Images were collected from each relevant area across a minimum of three cryosections per mouse. High magnification images were collected using the 40x air or 60x water objectives.

### Statistical Analysis

Statistical analyses were performed in Microsoft Excel and GraphPad Prism. Data was analysed by performing a one-way ANOVA followed by a Bonferroni multiple comparisons post-hoc test. Data is presented as mean ± standard deviation (std dev) unless otherwise stated and analyses were performed in n ≥ 3 mice in each case.

## Results

### *Pdgfrα-CreER*^*T2*^ transgenic mice can be used to specifically and efficiently induce recombination in OPCs in the CNS

**Fig 1:**
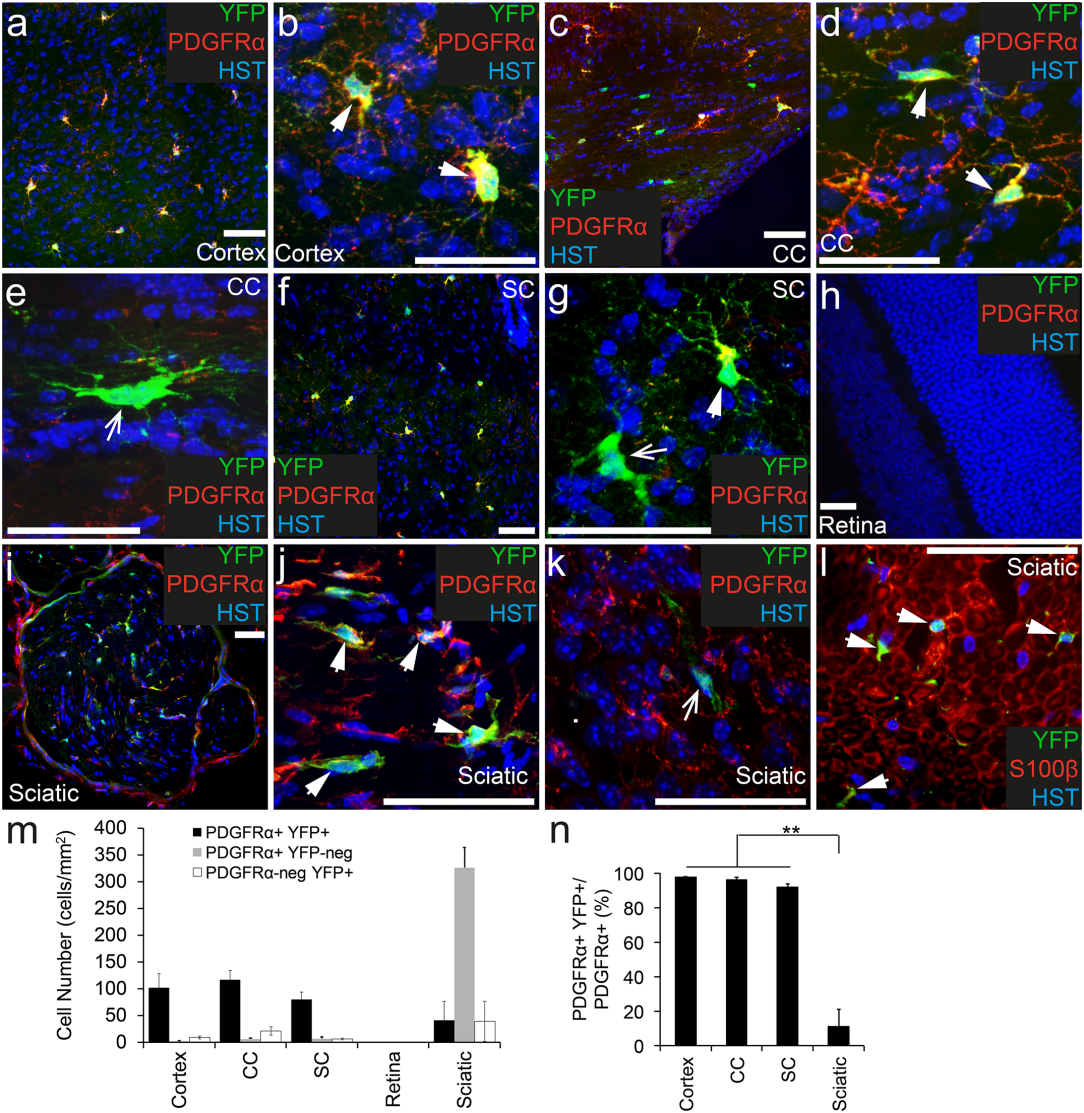
PDGFRα^+^ OPCs become YFP-labelled throughout the nervous system of adult *Pdgfrα-CreER*^*T2*^::*Rosa26-YFP* transgenic mice. Cryosections from P57+7 *Pdgfrα-CreER*^*T2*^::*Rosa26-YFP* transgenic mice were immunostained to detect PDGFRα (red), YFP (green) and Hoescht 33342 (HST, blue). Confocal image (single z plane) of the motor cortex at low (a) and high (b) magnification. Confocal image (single z plane) of the corpus callosum (cc) at low (c) and high (d, e) magnification. Confocal image (single z plane) of a transverse spinal cord section at low (f) and high (g) magnification. Confocal image (single z plane) of the retina (h). Confocal image (single z plane) of a transverse sciatic nerve section at low (i) and high (j, k) magnification. Cryosection from P57+7 *Pdgfrα-CreER*^*T2*^::*Rosa26-YFP* transgenic mouse immunostained to detect S100β (red), YFP (green) and HST (blue) (l). m) The number of PDGFRα^+^ YFP^+^, PDGFRα^+^ YFP-negative and PDGFRα-negative YFP^+^ cells quantified from confocal images (single z plane) of nervous system tissue, expressed as the number of cells per mm^2^. n) The proportion of PDGFRα^+^ cells that become YFP-labelled (PDGFRα^+^ YFP^+^ cells / total PDGFRα^+^ cells x 100) quantified from confocal images (single z plane) of nervous system tissue. Error bars represent mean ± std dev from n = 3 mice. ** P<0.001, ANOVA. PDGFRα^+^ YFP^+^ cells (and S100β^+^ YFP^+^ cells) are denoted by arrowheads. PDGFRα-negative YFP^+^ cells are denoted by arrows. Scale bars represent 50μm.

To confirm that OPCs are the only cell type to undergo recombination in the CNS of *Pdgfrα-CreER*^*T2*^ transgenic mice, adult *Pdgfrα-CreER*^*T2*^::*Rosa26-YFP* transgenic mice were treated with Tamoxifen, and perfusion fixed 7 days later (P57+7). The brain, spinal cord, retina and optic nerve were cryosectioned and immunohistochemistry performed to detect cells expressing PDGFRα (red), YFP (anti-GFP, green) and the nuclear marker Hoescht 33342 (Fig 1). PDGFRα^+^ OPCs were found throughout the CNS (Fig 1a–1g) with the exception of the retina (Fig 1h). The density of PDGFRα^+^ OPCs was equivalent in the cortex (104 ± 27 cells/mm^2^), corpus callosum (123 ± 20 cells/mm^2^) and spinal cord (87 ± 17 cells/mm^2^) (P>0.9 ANOVA; Fig 1m). The vast majority of OPCs became YFP-labelled in the CNS (Fig 1n) allowing clear visualization of their typical stellate morphology in the cortex (Fig 1b), corpus callosum (Fig 1d) and spinal cord (Fig 1g). Few PDGFRα^+^ OPCs escaped recombination as ~97% of PDGFRα^+^ OPCs were YFP-labelled in the cortex, ~96% in the corpus callosum, ~92% in the spinal cord (Fig 1n) and ~97% in the optic nerve (75 of 77 YFP^+^ cells counted). The retina lacked PDGFRα^+^ OPCs, and no YFP-labelled cells were detected (Fig 1h and 1m).

In addition to YFP-labelled OPCs, we identified a number of YFP^+^ cells that were PDGFRα-negative in the brain (Fig 1e) and spinal cord (Fig 1g), and these cells had the morphological characteristics of oligodendrocytes. Given that OPCs are known to give rise to new oligodendrocytes, producing new differentiated PDGFRα-negative cells even within 1 week of labelling [16], this was to be expected. The oligodendroglial identity of the YFP^+^ PDGFRα-negative cells was confirmed by performing immunohistochemistry to detect YFP and the oligodendrocyte lineage marker OLIG2. In the corpus callosum all YFP^+^ cells were also OLIG2^+^ GFAP-negative (639 YFP^+^ cells analyzed), confirming that the YFP^+^ PDGFRα-negative cells identified in this region were indeed oligodendrocytes. In the cortex, essentially all YFP^+^ cells were OLIG2^+^, with the exception of very rare YFP^+^ cells that instead co-labelled for the neuronal marker, NeuN (2 of 2035 YFP^+^ cells analyzed). A similarly small number of cortical neurons are GFP labelled in *Pdgfrα-histoneGFP* knock-in mice, despite their lack of PDGFRα expression [25]. Therefore, the YFP^+^ NeuN^+^ cells that we observe may be rare neurons that experience *Pdgfrα* gene promoter activity, but no PDGFRα protein translation.

These experiments confirm that the *Pdgfrα-CreER*^*T2*^ transgene is expressed by OPCs, and that Tamoxifen administration efficiently leads to DNA recombination in these cells.

### A small number of PDGFRα^+^ Schwann cells undergo recombination in the sciatic nerve of *Pdgfrα-CreER*^*T2*^::*Rosa26-YFP* transgenic mice

To determine whether cells within the peripheral nervous system express PDGFRα and / or undergo recombination in adult *Pdgfrα-CreER*^*T2*^::*Rosa26-YFP* transgenic mice, we processed cryosections from the sciatic nerve to detect PDGFRα (red), YFP (anti-GFP, green) and Hoescht 33342 (Fig 1i–1k). PDGFRα^+^ cells were detected throughout the sciatic nerve, and at the edge of each nerve bundle (Fig 1i). Surprisingly, the density of PDGFRα^+^ cells was significantly higher in the sciatic nerve (367 ± 17 cells/mm^2^) than any of the CNS regions examined (P<0.0001, ANOVA; Fig 1m). However, only ~11% of the PDGFRα^+^ cell population found in the sciatic nerve was YFP-labelled (Fig 1n). Furthermore, only half of the YFP^+^ cells examined co-labelled for PDGFRα (Fig 1k and 1m; 52% ± 5.7% of YFP^+^ cells co-expressed PDGFRα), indicating that the *Pdgfrα-CreER*^*T2*^ transgene also induced recombination in a small number of unidentified cells in the peripheral nervous system. Some Schwann cells in the sciatic nerve have been shown to express PDGFRα [26], and by performing immunohistochemistry to detect YFP (green) and the Schwann cell marker S100β (red; Fig 1l), we determined that all of the YFP^+^ cells present in the sciatic nerve of adult *Pdgfrα-CreER*^*T2*^::*Rosa26-YFP* transgenic mice were Schwann cells.

### Fewer than 1% of PDGFRα^+^ cells become YFP-labelled in the endocrine glands of adult *Pdgfrα-CreER*^*T2*^::*Rosa26-YFP* transgenic mice

**Fig 2:**
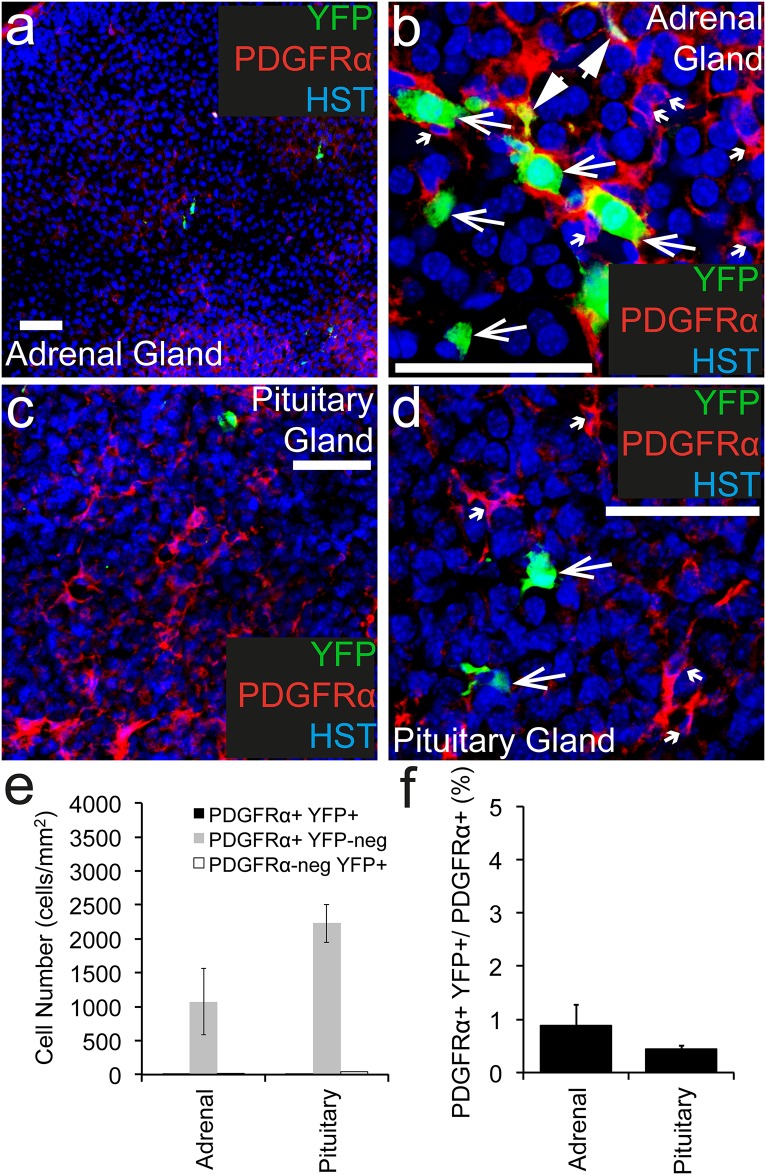
PDGFRα^+^ cells recombine at low efficiency in endocrine tissue. Cryosections through the adrenal and pituitary gland of P57+7 *Pdgfrα-CreER*^*T2*^::*Rosa26-YFP* were immunostained to detect PDGFRα (red), YFP (green) and Hoescht 33342 (blue). Confocal image (single z plane) of the adrenal gland at low (a) and high (b) magnification. Confocal image (single z plane) of the pituitary gland at low (c) and high (d) magnification. (e) The number of PDGFRα^+^ YFP^+^, PDGFRα^+^ YFP-negative and PDGFRα-negative YFP^+^ cells quantified from confocal images (single z plane) of the adrenal and pituitary glands, expressed as the number of cells per mm^2^. (f) The proportion of PDGFRα^+^ cells that become YFP-labelled (PDGFRα^+^ YFP^+^ cells / total PDGFRα^+^ cells, x 100) quantified from confocal images (single z plane) of adrenal and pituitary gland. Error bars represent mean ± std dev from n = 3 mice. PDGFRα^+^ YFP^+^ cells are denoted by arrowheads. PDGFRα-negative YFP^+^ cells are denoted by arrows. PDGFRα^+^ YFP-negative cells are denoted by small arrows. Scale bars represent 50μm.

To determine whether Tamoxifen administration to adult *Pdgfrα-CreER*^*T2*^::*Rosa26-YFP* transgenic mice can induce YFP-labelling in PDGFRα^+^ cells within the endocrine system, cryosections of the adrenal and pituitary glands were examined following immunohistochemistry to detect PDGFRα^+^ (red), YFP (green) and the nuclear marker Hoescht 33342 (Fig 2a–2d). PDGFRα^+^ cells were present throughout the adrenal (Fig 2a and 2b) and pituitary (Fig 2c and 2d) glands at a density of 1086 ± 500 cells/mm^2^ and 2237 ± 282 cells/mm^2^ respectively (Fig 2e). Of these PDGFRα^+^ cell populations, only ~0.9% and ~0.4% became YFP-labelled, respectively (Fig 2f). In addition to the small number of YFP^+^ PDGFRα^+^ cells detected, a number of YFP^+^ PDGFRα-negative cells were identified. In the adrenal gland these cells appeared to be clustered (Fig 2b), whereas in the pituitary gland they were more evenly distributed throughout the tissue (Fig 2d), but were still very rare. The YFP^+^ PDGFRα-negative cells represented 46% ± 24% of the YFP^+^ cells present in the adrenal gland and 81% ± 2.6% of the YFP^+^ cells in the pituitary gland. These data indicate that while the extent of recombination and YFP-labelling in the adrenal or pituitary glands of *Pdgfrα-CreER*^*T2*^::*Rosa26-YFP* mice is extremely low, the specificity of this labelling is also poor.

### Less than 1.5% of PDGFRα^+^ cells in the heart or gastrocnemius muscle undergo recombination following tamoxifen administration to adult *Pdgfrα-CreER*^*T2*^::*Rosa26-YFP* transgenic mice

**Fig 3:**
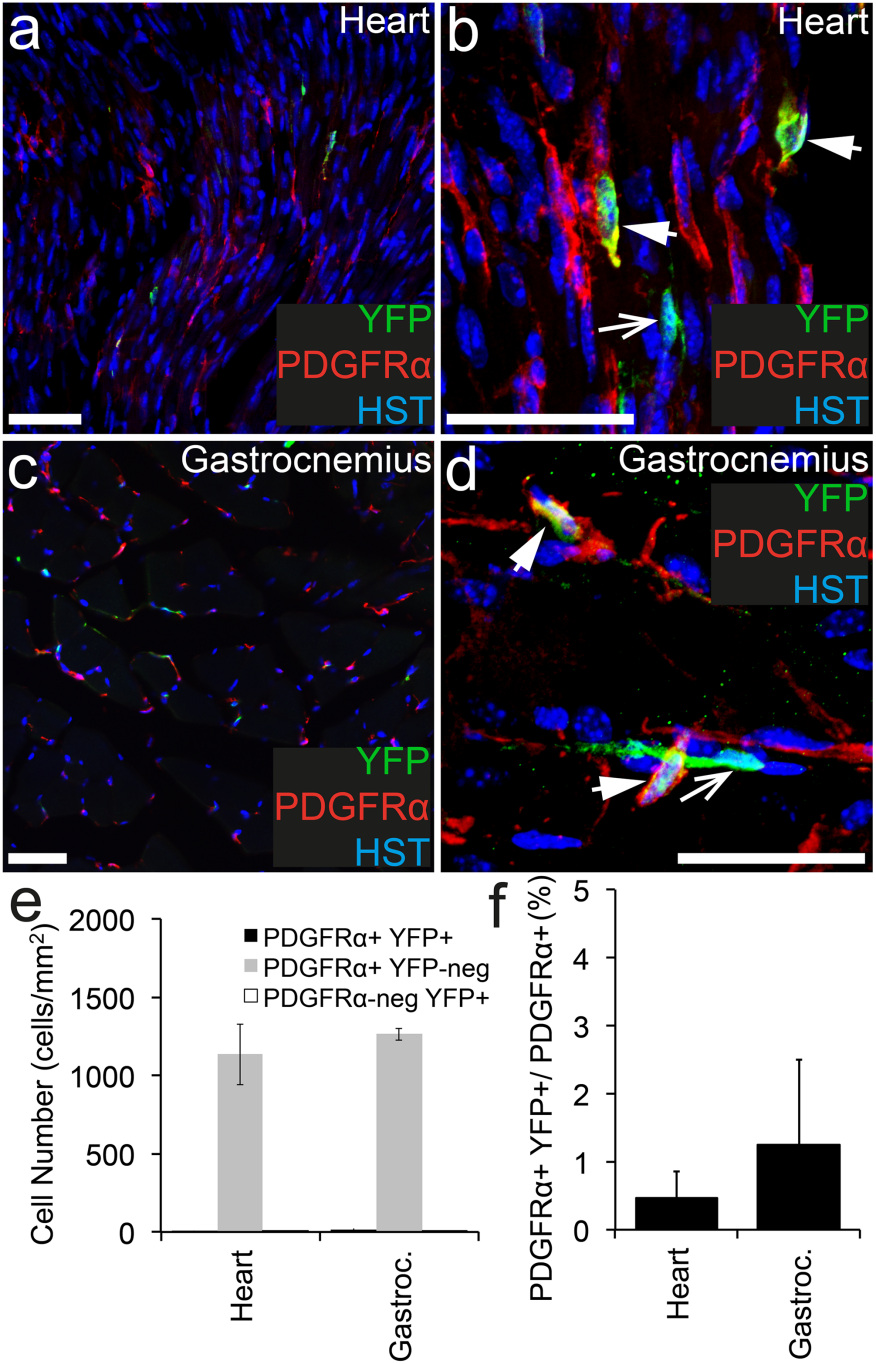
PDGFRα^+^ cells recombine at low efficiency in cardiac and skeletal muscle. Cryosections from P57+7 *Pdgfrα-CreER*^*T2*^::*Rosa26-YFP* transgenic mice were immunostained to detect PDGFRα (red), YFP (green) and HST (blue). Confocal image (single z plane) of the heart at low (a) and high (b) magnification. Confocal image (single z plane) of the gastrocnemius at low (c) and high (d) magnification. (e) The number of PDGFRα^+^ YFP^+^, PDGFRα^+^ YFP-negative and PDGFRα-negative YFP^+^ cells quantified from confocal images (single z plane) of the heart and gastrocnemius expressed as the number of cells per mm^2^. (f) The proportion of PDGFRα^+^ cells that become YFP-labelled (PDGFRα^+^ YFP^+^ cells/ total PDGFRα^+^ cells, expressed as a percentage) quantified from confocal images (single z plane) of the heart and gastrocnemius. Error bars represent mean ± std dev from n = 3 mice. PDGFRα^+^ YFP^+^ cells are denoted by arrowheads. PDGFRα-negative YFP^+^ cells are denoted by arrows. Scale bars represent 50μm.

To determine whether cells within the heart or gastrocnemius muscle become YFP-labelled in adult *Pdgfrα-CreER*^*T2*^::*Rosa26-YFP* transgenic mice, cryosections of each were stained to detect PDGFRα^+^ (red), YFP (green) and the nuclear marker Hoescht 33342 (Fig 3a–3d). PDGFRα^+^ cell populations were identified in the heart (Fig 3a and 3b) and gastrocnemius muscle (Fig 3c and 3d) at a similar density (Fig 3e) and these cells had a similar morphology (compare Fig 3b and 3d). The location and morphology of the PDGFRα^+^ cells in the heart and gastrocnemius muscle, are consistent with that of PDGFRα^+^ cardiac progenitor [27] and PDGFRα^+^ fibro/adipogenic progenitors (FAPs) [28] previously characterized. In the heart, only ~0.5% of the PDGFRα^+^ cell population became YFP-labelled (Fig 3a, 3b and 3f), which was not dissimilar to the ~1.2% of PDGFRα^+^ cells that became YFP-labelled in the gastrocnemius muscle (Fig 3a–3d and 3f). These data indicate that the *Pdgfrα-CreER*^*T2*^ transgenic mouse does not enable significant recombination in either of these cell populations.

In addition to the YFP^+^ PDGFRα^+^ cells identified in the heart and gastrocnemius muscle, we detected YFP^+^ PDGFRα-negative cells (Fig 3a and 3d). In the heart only 44% ± 6.3% of the YFP^+^ cells expressed PDGFRα, whereas in the gastrocnemius muscle, recombination was more specific with 83% ± 25% of the YFP+ cells co-expressing PDGFRα (Fig 3e). These data suggest that the *Pdgfrα-CreER*^*T2*^ transgene is ectopically expressed by some cells in the heart. However the extremely low number of YFP^+^ cells detected in either muscle suggests that the *Pdgfrα-CreER*^*T2*^ transgenic mouse is largely ineffective in targeting cells in these tissues.

### PDGFRα^+^ cells are present in the liver, lung, spleen and kidney, and recombine at low efficiency in adult *Pdgfrα-CreER*^*T2*^::*Rosa26-YFP* transgenic mice

**Fig 4:**
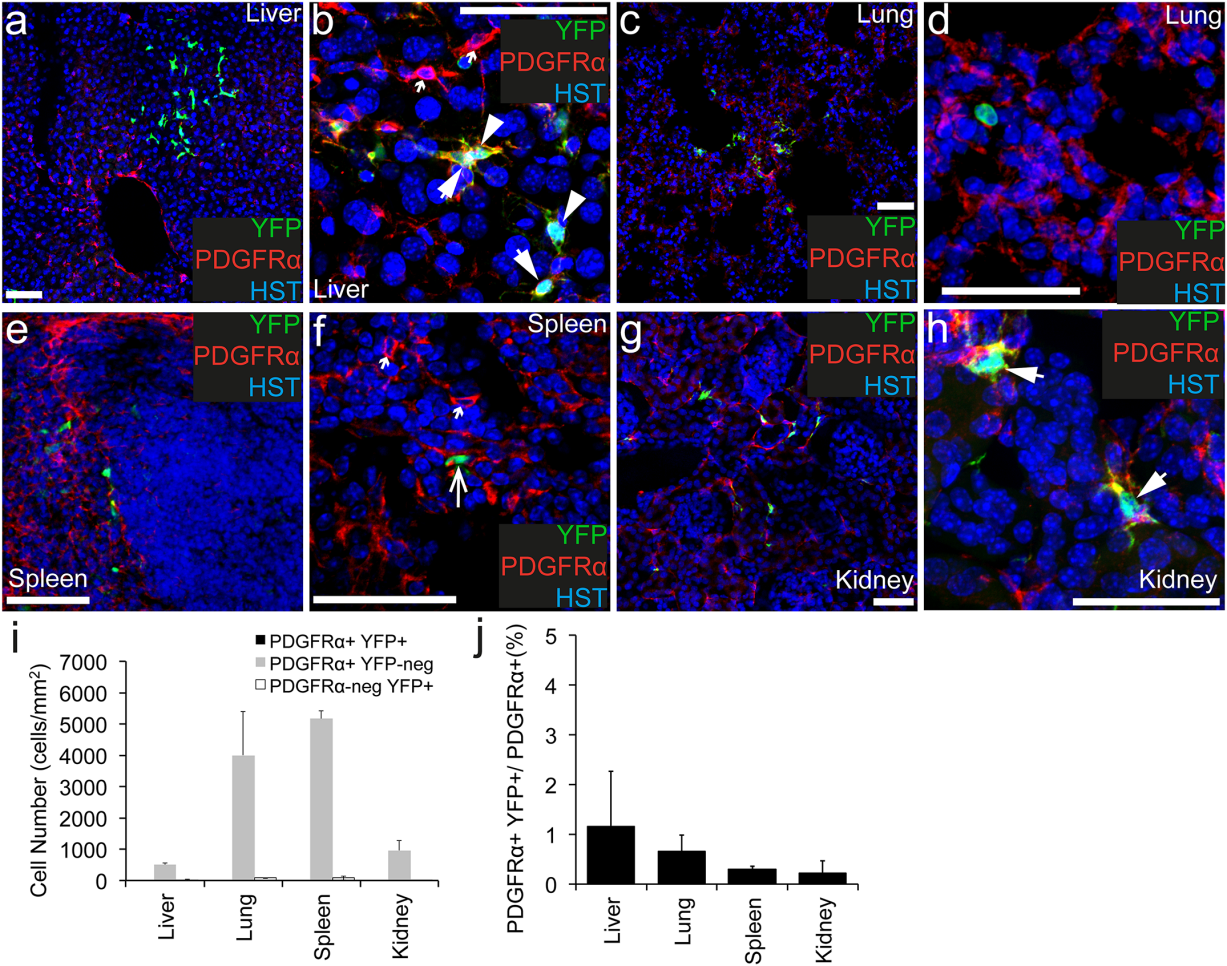
PDGFRα^+^ cells recombine at low efficiency in the liver, lung, spleen and kidney. Cryosections from P57+7 *Pdgfrα-CreER*^*T2*^::*Rosa26-YFP* transgenic mice were immunostained to detect PDGFRα (red), YFP (green) and HST (blue). Confocal image (single z plane) of the liver at low (a) and high (b) magnification. Confocal image (single z plane) of the lung at low (c) and high (d) magnification. Confocal image (single z plane) of the spleen at low (e) and high (f) magnification. Confocal image (single z plane) of the kidney at low (g) and high (h) magnification. (i) The number of PDGFRα^+^ YFP^+^, PDGFRα^+^ YFP-negative and PDGFRα-negative YFP^+^ cells quantified from confocal images (single z plane) of the liver, lung, spleen and kidney expressed as the number of cells per mm^2^. (j) The proportion of PDGFRα^+^ cells that become YFP-labelled (PDGFRα^+^ YFP^+^ cells/ total PDGFRα^+^ cells, expressed as a percentage) quantified from confocal images (single z plane) of the liver, lung, spleen and kidney. Error bars represent mean ± std dev from n = 3 mice. PDGFRα^+^ YFP^+^ cells are denoted by arrowheads. PDGFRα-negative YFP^+^ cells are denoted by arrows. PDGFRα^+^ YFP-negative cells are denoted by small arrows. Scale bars represent 50μm

To determine whether cells within the liver, lung, spleen or kidney undergo recombination and become YFP-labelled in adult *Pdgfrα-CreER*^*T2*^::*Rosa26-YFP* transgenic mice, cryosections were processed to detect PDGFRα^+^ (red), YFP (green) and the nuclear marker Hoescht 33342 (Fig 4). PDGFRα^+^ cells were dispersed throughout the liver (Fig 4a and 4b), lung (Fig 4c and 4d), spleen (Fig 4e and 4f) and kidney (Fig 4g and 4h). They were particularly numerous in the lung (4023 ± 1400 cells/ mm^2^) and spleen (5189 ± 236 cells/mm^2^). Despite each organ containing a higher density of PDGFRα^+^ cells than any CNS region examined (P<0.0003, ANOVA; Fig 4i), the number of YFP^+^ cells detected was extremely low. In the liver only ~1.2% of the PDGFRα^+^ cell population expressed YFP (Fig 4b and 4j). This was even lower in the other organs, with ~0.7% of the PDGFRα^+^ cells in the lung, ~0.3% of the PDGFRα^+^ cells in the spleen and ~0.2% of the PDGFRα^+^ cells in the kidney being YFP-labelled (Fig 4j). The YFP^+^ PDGFRα^+^ cells in the liver (Fig 4b) and kidney (Fig 4h) have a distinct fibroblast-like morphology. This is entirely consistent with previous reports that tissue fibroblasts express PDGFRα [29–32], and more specifically that hepatic stellate cells express PDGFRα in the adult liver [31,32]. Similarly, in the lung the YFP^+^ PDGFRα^+^ cells were detected in the alveoli (Fig 4g), consistent with previous reports that the PDGFRα^+^ lung cells are a subpopulation of lung fibroblasts called alveolar fibroblasts [29].

Only 18.8% ± 13% of the YFP^+^ cells in the liver, 22.9% ± 0.9% of the YFP^+^ cells in lung, 22% ±17% of the YFP^+^ cells in the spleen and 44% ± 3.8% of the YFP^+^ cells in the kidney co-expressed PDGFRα (Fig 4i). In the liver, the YFP^+^ cells (both YFP^+^ PDGFRα^+^ as well as YFP^+^ PDGFRα-negative cells) were unusually distributed—being rare overall, but when they were detected, they always appeared in clusters (as shown in Fig 4a). It is unlikely that the YFP^+^ PDGFRα-negative cells detected in the liver, lung, spleen and kidney are the result of the proliferation and differentiation of the YFP^+^ PDGFRα^+^ cells, as this would require each YFP^+^ PDGFRα^+^ cell to undergo between one and three cell divisions within the seven day labelling and tracing period, which is well above the level of cellgenesis previously reported in these tissues [33–35]. The more likely explanation is that the majority of YFP-labelling that occurs in these organs is non-specific. We conclude that the *Pdgfrα-CreER*^*T2*^::*Rosa26-YFP* transgenic mice cannot be used to specifically or efficiently induce gene recombination in the PDGFRα^+^ fiboblast-like cell populations in any of these organs.

### PDGFRα^+^ cells are present in the large and small intestine, and a small proportion of them become YFP-labelled in adult *Pdgfrα-CreER*^*T2*^::*Rosa26-YFP* transgenic mice

**Fig 5:**
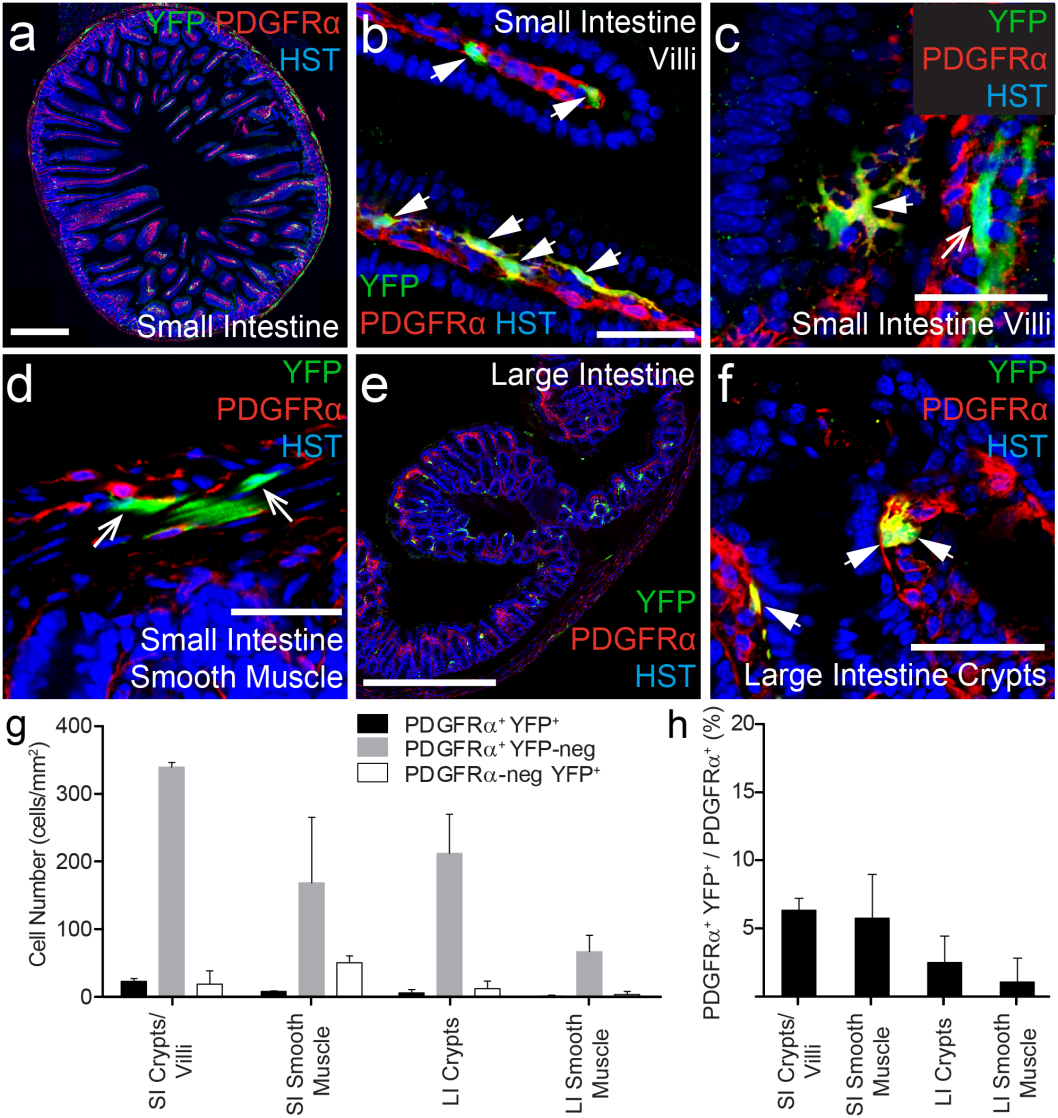
A small proportion of PDGFRα^+^ cells become YFP-labelled in the intestine. Cryosections from P57+7 *Pdgfrα-CreER*^*T2*^::*Rosa26-YFP* transgenic mice were immunostained to detect PDGFRα (red), YFP (green) and HST (blue). Confocal images (single z plane) of the small intestine at low (a) and high (b-d) magnification. Confocal images (single z plane) of the large intestine at low (e) and high (f) magnification. (g) The number of PDGFRα^+^ YFP^+^, PDGFRα^+^ YFP-negative and PDGFRα-negative YFP^+^ cells quantified from confocal images (single z plane) of the small (SI) and large (LI) intestine expressed as the number of cells per mm^2^. (h) The proportion of PDGFRα^+^ cells that become YFP-labelled (PDGFRα^+^ YFP^+^ cells / total PDGFRα^+^ cells, expressed as a percentage) quantified from confocal images (single z plane) of the small and large intestine. Error bars represent mean ± std dev from n = 3 mice. PDGFRα^+^ YFP^+^ cells are denoted by arrowheads. PDGFRα-negative YFP^+^ cells are denoted by arrows. Scale bars in (a) and (e) represent 500μm, All other scale bars represent 50μm.

The gastrointestinal tract is organized into distinct layers. A special population of PDGFRα^+^ cells, with a largely unknown function, has been previously identified within the mucosal layer of the intestine [36]. PDGFRα^+^ cells are also present in the muscular layer of the intestine where they mediate communication between the enteric nervous system and smooth muscle cells [37,38]. To determine whether these cells undergo recombination and become YFP-labelled in adult *Pdgfrα-CreER*^*T2*^::*Rosa26-YFP* transgenic mice, cryosections were processed to detect PDGFRα^+^ (red), YFP (green) and the nuclear marker Hoescht 33342 (Fig 5). We confirm that PDGFRα^+^ cells are present throughout the small intestine. In particular there was a high density of PDGFRα^+^ cells detected in the lamina propria of the villi (Fig 5a–5c and 5g). PDGFRα^+^ cells were also detected in the smooth muscle layer (Fig 5d), but no PDGFRα^+^ were detected in the epithelial cell layer (Fig 5b). In the large intestine, PDGFRα^+^ cells were similarly observed in the crypts and smooth muscle (Fig 5e and 5f). However, PDGFRα^+^ cell density was significantly higher in the small (344.3 ± 10 cells/mm^2^) relative to the large (184.9 ± 44.6 cells/mm^2^) intestine (P<0.002, ANOVA).

We next determined that YFP^+^ PDGFRα^+^ cells were present in the small and large intestines, however the proportion of PDGFRα^+^ cells that became YFP-labelled was quite small. Only ~6.4% of the PDGFRα^+^ cells in the villi and crypts of the small intestine and ~5.7% of the PDGFRα^+^ cells in the smooth muscle layer of the small intestine became YFP-labelled. Similarly, only ~2% of the PDGFRα^+^ cells in the large intestine co-expressed YFP (Fig 5g). Furthermore, the specificity of the YFP labelling in the intestine may also be of concern. In the villi and crypts of the small intestine 63% ± 22% of the YFP^+^ cells co-expressed PDGFRα (Fig 5b, 5c and 5g). However in the smooth muscle layer only 14% ± 3% of the YFP^+^ cells co-labelled for PDGFRα^+^ (Fig 5g). The YFP^+^ PDGFRα-negative cells in the smooth muscle layer were elongated and had long narrow nuclei, suggesting that they were smooth muscle cells. Therefore, while the *Pdgfrα-CreER*^*T2*^ transgenic mouse can be used to label a small subpopulation of the intestinal PDGFRα^+^ cell population for lineage tracing studies, it would be ineffective for facilitating conditional gene deletion in these cells.

### PDGFRα^+^ cells are present in the bone marrow and become YFP-labelled following Tamoxifen administration to adult *Pdgfrα-CreER*^*T2*^::*Rosa26-YFP* transgenic mice

**Fig 6:**
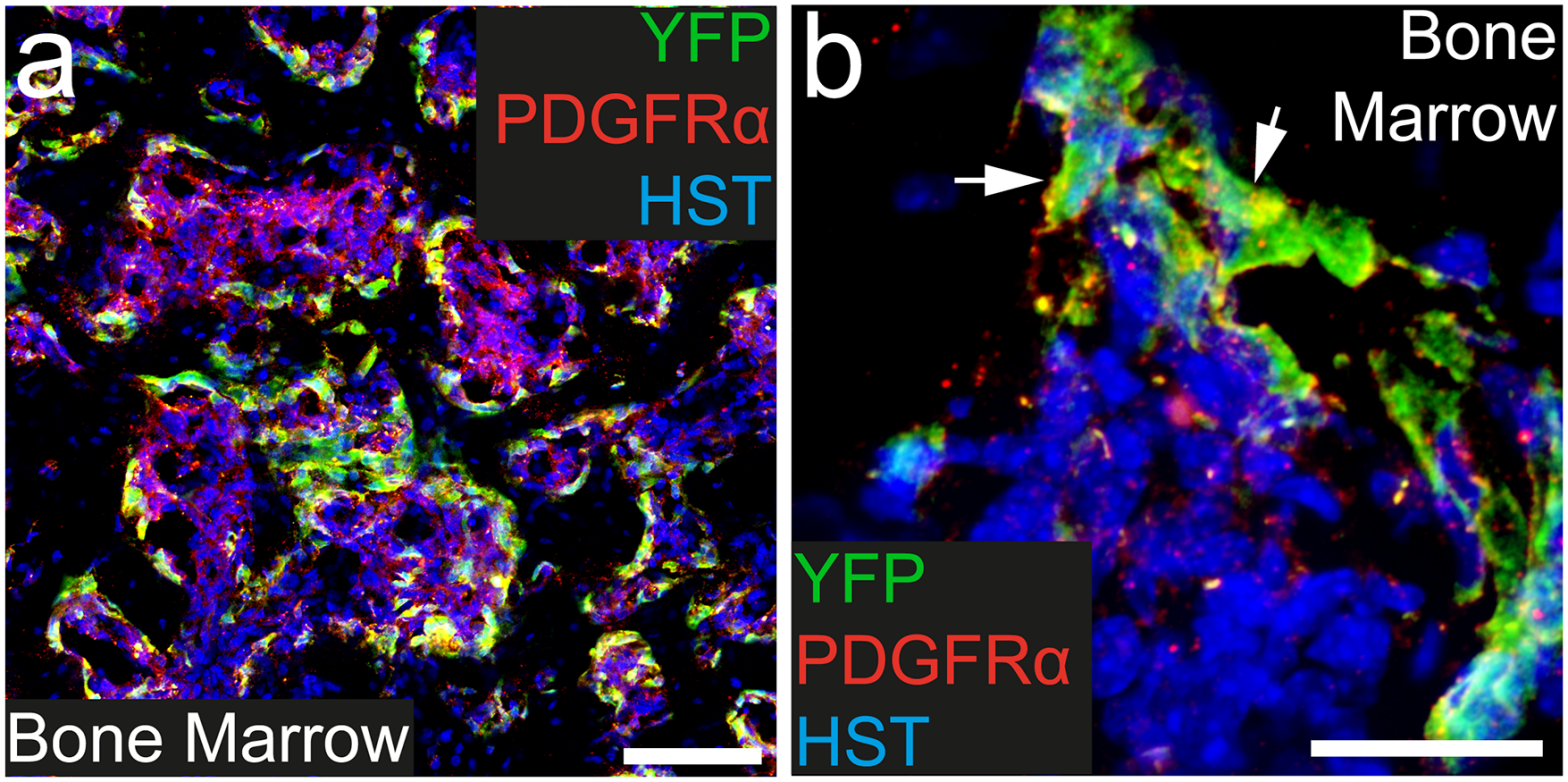
A proportion of PDGFRα^+^ cells become YFP- labeled in the bone marrow. Transverse cryosections taken through the tail of P57+7 *Pdgfrα-CreER*^*T2*^::*Rosa26-YFP* transgenic mice were immunostained to detect PDGFRα (red), YFP (green) and HST (blue). Confocal images (single z plane) of the bone marrow at low (a) and high (b) magnification. PDGFRα^+^ YFP^+^ cells are denoted by arrowheads. Scale bars represent 100μm (a) and 25μm (b).

Within the bone marrow PDGFRα^+^ cells are non-hematopoietic stromal cells [39]. To determine whether these PDGFRα^+^ cells become YFP labelled in adult *Pdgfrα-CreER*^*T2*^::*Rosa26-YFP* transgenic mice, cryosections were taken through the tail and processed to detect PDGFRα (red), YFP (green) and the nuclear marker Hoescht 33342 (Fig 6). As expected PDGFRα^+^ cells were present throughout the bone marrow (Fig 6a and 6b) at high density (3545.7 ± 619.5 cells/mm^2^). Furthermore, 37.9 ± 0.8% of these cells had undergone recombination and were YFP labeled. Within the bone marrow, the YFP-labelling was highly specific, with all YFP-labeled cells expressing PDGFRα^+^. These data indicate that, outside of the CNS, the most specific and extensive site of recombination and YFP-labelling achieved using the *Pdgfrα-CreER*^*T2*^::*Rosa26-YFP* transgenic mice, is within the bone marrow.

## Discussion

### *Pdgfrα-CreER*^*T2*^ transgenic mice specifically and efficiently induce recombination in OPCs in the CNS

Within the CNS, PDGFRα and the NG2 proteoglycan are both accepted markers for the identification of OPCs, as they are co-expressed by >98% of OPCs [16]. While inconsistent with the protein expression data, *Ng2-dsred* transgenic mice not only label OPCs within the CNS, but also robustly label pericytes [40], whereas *Pdgfrα-CreER*^*T2*^ transgenic mice have been used to induce cre-mediated recombination soley within OPCs [16,19]. In this study we report that *Pdgfrα-CreER*^*T2*^ transgenic mice can induce recombination in >90% of OPCs across the CNS, ranging from ~91% in the spinal cord to ~97% in the motor cortex (see Fig 1). These data are consistent with previous reports in which the administration of Tamoxifen to adult *Pdgfrα-CreER*^*T2*^::*Rosa26-mGFP* transgenic mice resulted in the GFP-labelling of ~94% of OPCs in the dorsal cortex and ~91% of OPCs in the piriform cortex [41]. Essentially all PDGFRα-negative YFP^+^ cells observed in the CNS had a distinct oligodendrocyte morphology and co-labelled for OLIG2, suggesting that they were newly differentiated oligodendrocytes, generated by the YFP-labelled OPCs. More of these cells were observed in the corpus callosum than the cortex, which is also consistent with previous observations that OPCs within the adult mouse corpus callosum proliferate and generate new oligodendrocytes more rapidly than those in the cortex [16].

We conclude that within the CNS, expression of the *Pdgfrα-CreER*^*T2*^ transgene faithfully follows that of endogenous PDGFRα expression, in that it specifically facilitates DNA recombination in a high proportion of OPCs. This transgenic mouse can therefore be used to effect gene activation or deletion within OPCs of the CNS, without affecting other CNS cell types. However, its suitability for some experiments, particularly gene deletion experiments, is also dependent on the level of recombination (or lack of recombination) achieved in cells outside of the CNS.

### The *Pdgfrα-CreER*^*T2*^ transgene enables recombination in a small proportion of Schwann cells

We have determined that a small proportion (~11%) of PDGFRα^+^ S100β^+^ Schwann cells, and a small number of PDGFRα-negative S100β^+^ Schwann cells, become YFP-labelled in P57+7 *Pdgfrα-CreER*^*T2*^::*Rosa26-YFP* transgenic mice (see Fig 1). This reflects a low recombination efficiency for the Schwann cell population overall. However since the vast majority of peripheral myelin is laid down by P22 [42], and only a small number of Schwann cells are still adding myelin at P57 [42], it is possible that the less mature cells are preferentially labelled using the *Pdgfrα-CreER*^*T2*^ transgenic mouse. Irrespective of their stage of maturation, this is the first report of Schwann cell labelling in the sciatic nerve of *Pdgfrα-CreER*^*T2*^ mice. A previous study, using an alternative *Pdgfrα-CreER*^*T2*^ mouse line, achieved recombination in the sciatic nerve following a crush injury, but the labelled cells were not Schwann cells [43]. However, given the small proportion of Schwann cells that became YFP-labelled in the *Pdgfrα-CreER*^*T2*^ mouse line used for this study, and the known capacity of peripheral nerves to regenerate following an injury [44,45], it is unlikely that using the *Pdgfrα-CreER*^*T2*^ transgenic mouse for conditional gene ablation, irrespective of the gene, would seriously impact peripheral nerve function or result in a detectable or long-lived Schwann cell phenotype.

### *Pdgfrα-CreER*^*T2*^ transgenic mice induce recombination in a novel PDGFRα^+^ cell population in the intestine

A unique population of PDGFRα^+^ cells reside in the mucosal layer of the gastrointestinal tract [36]. These cells have long, thin cell bodies and processes, and are found in the laminar propria where they form a sheath that extends along the length of the crypts, immediately below the epithelial cell layer. Many of the PDGFRα^+^ YFP^+^ cells detected in the villi and crypts of the small and large intestine of *Pdgfrα-CreER*^*T2*^::*Rosa26-YFP* transgenic mice belong to this population of cells. Interestingly, it has been suggested that these subepithelial PDGFRα^+^ cells may be able to remodel or change their phenotype depending upon cell culture conditions [36], and this could be a possible explanation for the occasional YFP^+^ PDGFRα^+^ cells in the villi of the small intestine that are morphologically distinct from most of this population (see Fig 5c). However, little is known about this newly discovered cell type and its *in vivo* differentiation capabilities.

In the muscle layers of the small and large intestine, PDGFRα^+^ cells are a subtype of interstitial cell, also known as “fibroblast-like” cells [37]. These cells are located near the terminals of enteric motor neurons and communicate information from the enteric nervous system to the smooth muscle cells of the intestine via gap junctions [37,38]. In *Pdgfrα-CreER*^*T2*^::*Rosa26-YFP* transgenic mice, Tamoxifen administration induced recombination and YFP-labelling in a very small proportion of PDGFRα^+^ cells in any muscle type. The intestinal smooth muscle had the highest level of recombination with ~5.7% of PDGFRα^+^ cells becoming YFP labeled, while in the cardiac and skeletal muscle <2% of PDGFRα^+^ cells recombined. Morphologically the PDGFRα^+^ cell population in the smooth muscle is similar to the PDGFRα^+^ cell populations identified in the heart and gastrocnemius muscle (compare Fig 3b and 3d with Fig 5d), which is particularly interesting as a number of studies have indicated that the PDGFRα^+^ cells in the heart [27,46] and skeletal muscle [47,48] are progenitor populations.

While few of the PDGFRα^+^ cells in the heart and small intestinal smooth muscle underwent recombination, these regions contained a relatively high proportion of YFP^+^ PDGFRα-negative cells. One possible explanation for these cells is that the YFP^+^ PDGFRα^+^ cells represent immature cell populations which, like the OPCs, rapidly divide and mature into cells that no longer express PDGFRα. Even though the PDGFRα^+^ cells in the heart are multipotent progenitors [46], their rate of proliferation and differentiation could not account for the number of YFP^+^ PDGFRα-negative cells detected [49]. Alternatively, in the intestinal smooth muscle, it is possible that YFP could pass from recombined YFP^+^ PDGFRα^+^ cells through the gap junctions to give the appearance of YFP-labelling in PDGFRα-negative cells. However, this seems unlikely as there does not appear to be a close physical association between these two cell populations. The most likely explanation is that the *Pdgfrα-CreER*^*T2*^ transgene lacks a regulatory element that controls the expression of *Pdgfrα* –one that is not required to regulate gene expression in the CNS, so that it is mis-expressed, resulting in the direct recombination of small numbers of PDGFRα-negative cells in these tissues.

### PDGFRα^+^ stromal cells in the bone marrow become YFP-labelled more efficiently than any other PDGFRα^+^ cell population outside of the CNS

The bone marrow is a major site of hematopoiesis. However, surrounding the islands of hematopoietic cells, is an extensive network of stromal cells that includes the PDGFRα^+^ cells of the bone marrow. The PDGFRα^+^ stromal cells comprise the perivascular CXCL12-abundant reticular (CAR) cells and the PDGFRα^+^ Sca1^+^ (PαS) cells [39]. CAR cells are adipo-osteogenic progenitors that secrete factors essential for haematopoietic stem and progenitor cell maintenance [50–52], while PαS cells are a population enriched in bone marrow mesenchymal stem cells [53]. Following Tamoxifen administration to adult *Pdgfrα-CreER*^*T2*^::*Rosa26-YFP* transgenic mice, ~38% of PDGFRα^+^ bone marrow stromal cells become YFP-labelled, and the recombination of cells in this region was highly specific, as all YFP^+^ cells were PDGFRα^+^. These data indicate, that the *Pdgfrα-CreER*^*T2*^ transgenic mouse induces a significant level of gene recombination in PDGFRα^+^ bone marrow stromal cells *in vivo*, and could be useful for lineage tracing studies of this population. Furthermore, if researchers plan to use *Pdgfrα-CreER*^*T2*^ transgenic mice to achieve conditional gene deletion from OPCs in the CNS, they should also consider that the gene will also be deleted from cells in this population.

### Few PDGFRα^+^ fibroblasts become YFP-labelled in *Pdgfrα-CreER*^*T2*^::*Rosa26YFP* transgenic mice

We identified PDGFRα^+^ cell populations in all of the tissues and quantified the level of recombination (YFP-labelling) achieved using *Pdgfrα-CreER*^*T2*^ transgenic mice. The majority of PDGFRα^+^ cell populations identified were fibroblasts [30]. PDGFRα is an established marker for some fibroblast cell populations. For example, *in situ* hybridisation previously indicated the presence of *Pdgfrα* mRNA in the lung [2], and a GFP reporter line was then used to visualize these cells [54], and determine that PDGFRα expression identifies alveolar fibroblasts [29]. Hepatic stellate cells, a fibrotic cell type in the liver, have also been shown to express PDGFRα [31,32]. Furthermore, the overexpression of PDGFRα has been linked to fibrosis in many of the organs examined in this study, such as the lung, kidney and liver [31,55]. In each of the tissues that contained PDGFRα^+^ fibroblasts, very few of these cells became YFP-labelled in P57+7 *Pdgfrα-CreER*^*T2*^::*Rosa26-YFP* transgenic mice. The low level of recombination in these populations and poor specificity for recombination in these areas, suggests that these mice will have limited application for studying these cell types. Furthermore, if researchers plan to use the *Pdgfrα-CreER*^*T2*^ transgenic mouse to achieve conditional gene deletion from OPCs in the CNS, they are unlikely to experience unexpected effects in the lungs, liver, kidney or spleen.

## Conclusions

*Pdgfrα-CreER*^*T2*^ transgenic mice are an effective tool for selectively achieving highly specific recombination in PDGFRα^+^ OPCs of the CNS. However, outside of the CNS, these mice fail to induce significant recombination in any PDGFRα^+^ cell population, with the exception PDGFRα^+^ stromal cells in the bone marrow.
